# Confinement in crystal lattice alters entire photocycle pathway of the Photoactive Yellow Protein

**DOI:** 10.1038/s41467-020-18065-9

**Published:** 2020-08-25

**Authors:** Patrick E. Konold, Enis Arik, Jörn Weißenborn, Jos C. Arents, Klaas J. Hellingwerf, Ivo H. M. van Stokkum, John T. M. Kennis, Marie Louise Groot

**Affiliations:** 1 Department of Physics and Astronomy and LaserLaB, Faculty of Science, Vrije Universiteit, De Boelelaan 1081, 1081 HV, Amsterdam, The Netherlands; 2 Laboratory for Microbiology, Swammerdam Institute for Life Sciences, University of Amsterdam, Science Park, 1098 XH Amsterdam, The Netherlands

**Keywords:** Proteins, Molecular conformation, X-ray crystallography, Reaction kinetics and dynamics

## Abstract

Femtosecond time-resolved crystallography (TRC) on proteins enables resolving the spatial structure of short-lived photocycle intermediates. An open question is whether confinement and lower hydration of the proteins in the crystalline state affect the light-induced structural transformations. Here, we measured the full photocycle dynamics of a signal transduction protein often used as model system in TRC, Photoactive Yellow Protein (PYP), in the crystalline state and compared those to the dynamics in solution, utilizing electronic and vibrational transient absorption measurements from 100 fs over 12 decades in time. We find that the photocycle kinetics and structural dynamics of PYP in the crystalline form deviate from those in solution from the very first steps following photon absorption. This illustrates that ultrafast TRC results cannot be uncritically extrapolated to in vivo function, and that comparative spectroscopic experiments on proteins in crystalline and solution states can help identify structural intermediates under native conditions.

## Abstract (web summary)

Protein structural dynamics can be studied by time-resolved crystallography (TRC) and ultrafast transient spectroscopic methods. Here, the authors perform electronic and vibrational transient absorption measurements to characterise the full photocycle of Photoactive Yellow Protein (PYP) both in the crystalline and solution state and find that the photocycle kinetics and structural intermediates of PYP deviate in the crystalline state, which must be taken into consideration when planning TRC experiments.

## Introduction

Time-resolved macromolecular crystallography (TRC) enables direct visualization of a protein structure while exerting its function, e.g., during signaling, ion translocation and catalysis^1–7^, and with the advent of free-electron lasers, timescales of femto- to picoseconds are now experimentally accessible^4,8–15^. Consequently, protein structural dynamics can now be studied with both TRC and various ultrafast transient spectroscopic methods. Vibrational spectroscopy offers, after successful assignment, high resolution structural information on interatomic distances and dihedral angles of catalytically active groups^16^. Therefore, the combination of TRC and spectroscopic techniques enables biophysical interrogation with the utmost resolution. A looming pitfall in this pursuit concerns the sample treatment in each method. Spectroscopy is routinely performed on proteins dissolved in aqueous buffer solution of known pH—largely consistent with their native state in vivo. For TRC in contrast, proteins are tightly packed in a regular lattice with lower relative hydration compared to the solvated state in vivo. Hence, the question arises how these conditions influence the (photo)chemical reactions and how the ensuing structural transformations vary within the two environments.

Here, we investigated the full photocycle dynamics of a small signal-transduction protein, Photoactive Yellow Protein (PYP), responsible for the negative phototactic response in the phototrophic bacterium *Halorhodospira halophila*^17^. This protein is an excellent system to study the effect of crystallization on experimental photo-transformations, as PYP is relatively small (14 kDa), biochemically stable, water-soluble, and is especially relevant because it has served as a model system for the earliest TRC studies^1–3,5,11,15^. We report electronic and vibrational transient absorption measurements spanning the full PYP photocycle in crystalline form and dissolved in buffer solution.

## Results

### Initiating the PYP photocycle

PYP has an intrinsic chromophore, *para*-hydroxycinnamic acid (pCa)—an extensively conjugated anion, which is covalently attached to a cysteine residue (Cys69) through a thioester linkage to the protein backbone^18^. Upon blue light absorption (*λ*_max_ = 446 nm), PYP enters a photocycle involving several intermediates with lifetimes ranging from femtoseconds to seconds, culminating in a large secondary structural change that underlies its signaling function. We initiated the photocycle by excitation of the pCa with a 50 fs laser pulse centered at 475 nm and recorded electronic transient absorption measurements from 100 fs to 0.3 ms using electronically-synchronized femtosecond lasers and augmented these data, with data from Yeremenko et al.^19^, collected from microseconds to 1 s, to span the full photocycle dynamics. We used a low excitation power of 400 nJ, focused to a spot size of 250 µm in diameter, which, centered on the flank of the absorption band of PYP, resulted in 5% excited proteins, thus avoiding multi-photon and/or photo-ionization processes^20^, for both PYP in the crystalline form (PYP_C_) and in solution (PYP_S_).

### Transients in the UV-visible spectral region

**Fig. 1:**
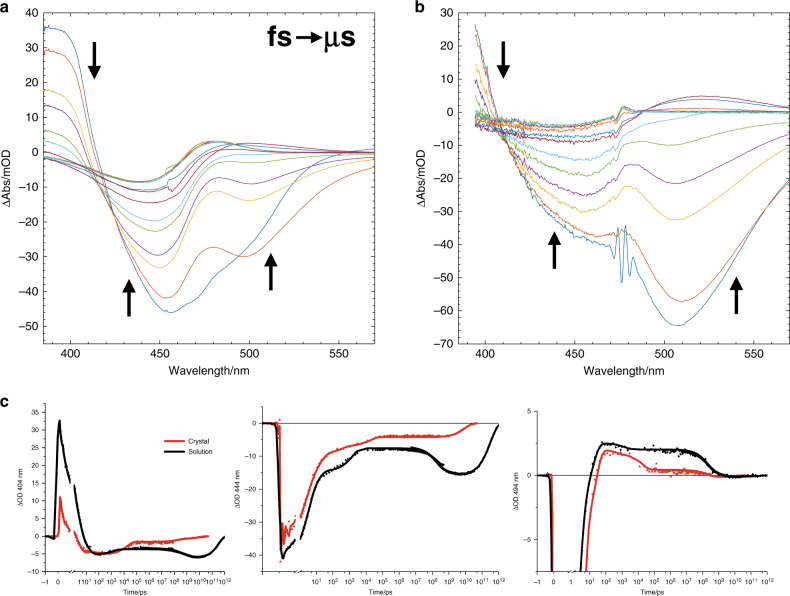
Transient absorption data in the visible collected over 12 decades in time. A selection of transient absorption spectra as a function of time delay are shown, upon excitation with a 50-fs laser pulse centered at 475 nm, for **a** PYP_S_ and **b** PYP_C_. The temporal evolution is indicated by the black arrows. In PYP_C_, a scattered-light artifact is visible at the wavelength of excitation. **c** Wavelength traces at 404, 444 and 494 nm of PYP_S_ (black) and PYP_C_ (red), see Supplementary Figs. 1 and 2 for traces at additional wavelengths. The data from the current study with sub-picosecond time resolution has been merged with data taken from Yeremenko et al.^19^ with microsecond time resolution, with the latest time point at 48 ms (crystal) or 1 s (solution). Wavelength is indicated in the ordinate label. Note that the time axis is linear until 1 ps (after the maximum of the instrument response function (IRF)), and logarithmic thereafter. A single scaling parameter has been used to connect the data from the experiments with sub-picosecond and microsecond time resolution. PYP_S_ was measured at pH 8, PYP_C_ at pH 6.5. PYP_S_ time traces collected at pH 6 are shown in Supplementary Fig. 1 and exhibit corresponding dynamics.

The electronic responses of PYP_C_ and PYP_S_ were recorded in the 380–570 nm spectral window, and primarily reveal photochemical changes to the pCa chromophore. Figure 1a, b shows a selection of the collected absorption difference spectra for PYP_S_ and PYP_C_ and panel C provides a more detailed look at the kinetics at three selected wavelengths, comparing the dynamics of PYP_S_ (black) and PYP_C_ (red). Additional time traces are shown in Supplementary Figs. 1 and 2. The time traces reported in Yeremenko et al.^19^ have been overlaid with those recorded here, which resulted in an excellent agreement between the two separate experiments in the overlap region between 10–300 µs.

Upon excitation, an immediate buildup of a mixed signal composed of excited state absorption (*λ*^max^ ≈ 400 nm), bleached ground state absorption (≈450 nm) and stimulated emission (≈500 nm) is observed. With increasing pump-probe delay time, the excited state and stimulated emission signals decay and product formation is observed in the 480–500 nm region. Then, on a sub-millisecond timescale, the product signal at ≈480–500 nm decays, the ground state bleach shows an increase in signal, due to disappearance of the compensating red-product absorption, and a small product band appears <400 nm (not fully covered in Fig. 1a, b). Notable differences between the traces of PYP_S_ and PYP_C_ exist on all timescales. For example, the dynamics on the (sub-)picosecond timescale appear different, with a faster initial decay in PYP_C_, while the yield of photoproduct formation in PYP_C_ is lower than in PYP_S_. Differing dynamics were also observed on the 10–20 ns timescale that is absent in PYP_S_. Spectral differences between the solution and crystalline samples are also present, these are most likely due to the higher absorption in the crystals, diminishing the bleach signals at 440 nm and to increased scatter, which leads to a certain degree of distortion of the absorption difference spectra. The steady state absorption spectra of PYP_S_ and PYP_C_ differ only slightly, crystalline red-shifted (3–4 nm) with a similar width and overall shape (Supplementary Fig. 3), in agreement with earlier observations^19,21,22^.

### Transients in the mid-IR spectral region

**Fig. 2:**
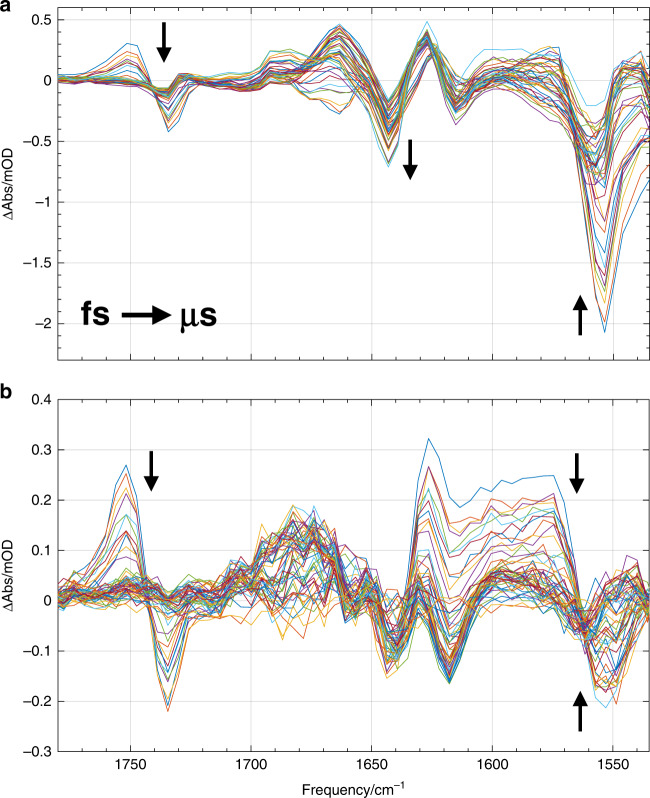
Transient absorption difference spectra in the midinfrared. A selection of transient absorption difference spectra in the mid-IR spectral region following excitation with 475 nm light for **a** PYP_S_ and **b** PYP_C_, from 100 fs to 100 ms. Full spectra were recorded for each laser pulse after dispersing the probe light on a 64 element Mercury Cadmium Telluride photodiode array, resulting in a ~4 cm^−1^ sampling resolution. Approximate temporal evolution from femtoseconds to microseconds is denoted by the black arrows.

We further recorded the absorption changes induced in the mid-IR region between 1780 and 1530 cm^−1^ upon excitation at 475 nm for PYP_S_ and PYP_C_ (Fig. 2). The vibrational response in the mid-IR region enables precise assignment of structural dynamics that occur throughout the photocycle; specifically, changes to the hydrogen bond network surrounding the chromophore, changes in the chromophore structure and concerted changes of the protein backbone.

**Fig. 3:**
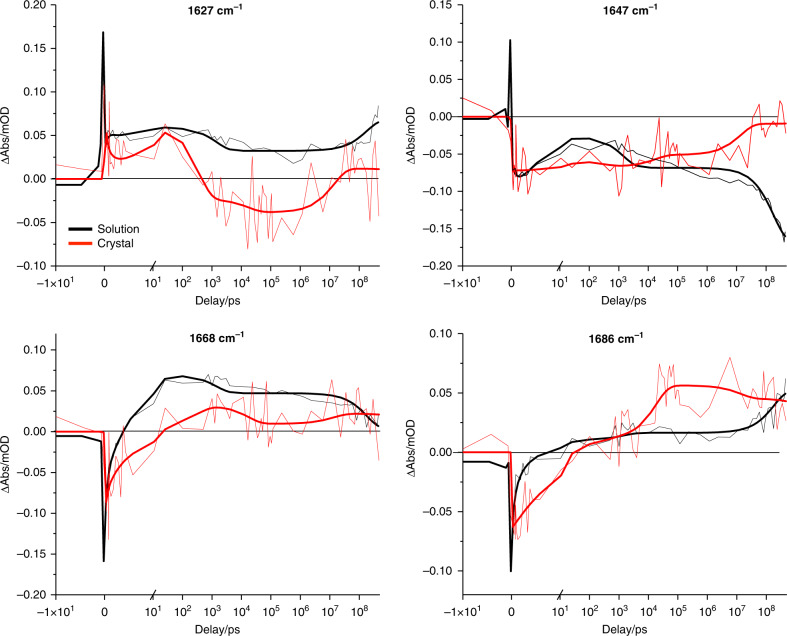
Transient absorption time traces in the midinfrared. Time traces at selected wavenumbers of PYP_S_ (black) and PYP_C_ (red). Note that the time axis is linear until 10 ps (after the maximum of the IRF), and logarithmic thereafter. The PYP_S_ data was scaled lower by a factor of 5 to facilitate comparison.

Time traces collected in the mid-IR region up to 0.3 ms show a similar divergence between the two PYP forms as in the visible spectral region. Figure 3 presents time traces at selected frequencies: 1668 cm^−1^, the dynamics forming a positive feature are delayed in the crystal with respect to solution, and at 1686 cm^−1^ the rise is more complex in the crystal than in solution. As will be argued in more detail below based on ^13^C isotope labeled measurements, these frequencies track the pCa carbonyl, and record the breaking of the hydrogen bond between the pCa carbonyl and Cys69, with its precise position being dependent on the environment of the carbonyl. See Supplementary Figs. 4–6 for a complete representation of the collected time traces.

### Photocycle analysis

**Fig. 4:**
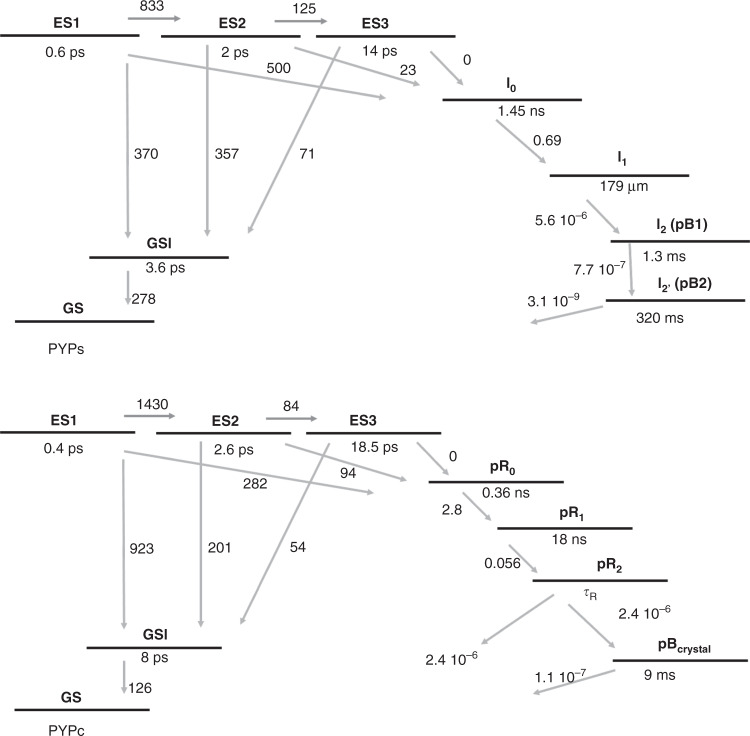
Kinetic schemes of the photocycle of PYP in solution and in crystalline form. Top panel: PYP in solution, bottom panel: PYP in crystalline form. Upon absorption of light by pCa in the ground state (GS), the excited state is formed from which the system transitions through the reaction. The relevant states are indicated by the following abbreviations: ES_1-3_: excited states sharing the same spectrum, evolving sequentially from 1→2→3; GSI: ground state intermediate; I_0_, I_1_, I_2_ (pB1), I_2’_(pB2), pR_0_, pR_1_, pR_2,_ pB_crystal_: photocycle intermediates. Note that we use the symbol “I_X_” for notation of PYP_S_ intermediate states, and “pC” (with C for color, i.e., red or blue) for those in PYP_C_, except for the final pB states. The transition rates indicated near the arrows are in ns^−1^, the lifetime of each state is indicated in italic. The rate constants involving the ES and GSI for PYP_S_ have been fixed from ref. ^24^, all other parameters have been estimated. Note that in the crystal as evidenced from the UV–Vis data^19^ half of the pR_2_ states is converted to pB, whereas the other half branches directly to the ground state, with a rate of k_R,VIS_ 2.4 × 10^−6^. Note that the longer time scales (after 100 ms) are based solely on the the UV–Vis data of ref. ^19^. Concentration profiles computed with these kinetic schemes for the photocycle intermediates states are shown in Supplementary Fig. 7.

To resolve the individual steps and intermediates in the PYP photocycle, all time traces were fitted to a target model^23–25^, analogous to models previously used for analysis of TRC dynamics^2,3,11^. Strikingly, we found that the visible and mid-IR data of PYP_S_ in solution could be described with the same model (Fig. 4), with excellent fit quality. This indicates that the electronic and vibrational absorption changes display identical dynamics, which enables direct comparison of the species associated difference spectra (SADS) obtained with these two types of spectroscopy. Likewise, the visible and mid-IR PYP_C_ data could be fitted with the same kinetic model. However, as compared to the PYP_S_ data, according to the divergent kinetics discussed above, different parameters were required to achieve an adequate fit for each PYP_S_ and PYP_C_, and, notably, the crystalline kinetic model included an additional intermediate state on the nanosecond time scale (Fig. 4).

Figure 4 shows the target models used for the solution and crystallized states. The nomenclature for PYP photocycle intermediates varies throughout the literature, and since distinct dynamics are observed here for PYP_C_, we use the symbol “I_X_” for notation of PYP_S_ intermediate states, and “pC” (with C for color, i.e., red or blue) for those in PYP_C_, except for the final pB states. Previous pump-dump-probe, transient absorption and fluorescence studies^24,26–34^ were used to define and confine our target model. With target analysis, one can extract the characteristic spectra of the photocycle states, namely, contributions from the excited state (ES), primary photoproduct (I_0_), and subsequent intermediates. The target model in Fig. 4 describes the experimentally observed key elements of the PYP photocycle^23,24,28,31,33–35^ such as the multi-phasic decay of the excited-state and the involvement of a ground state intermediate, and allows for estimation of the quantum yield of isomerization, which has earlier been found to be highest from the shorter-lived excited states^24^. The multi-phasic decay of the excited state is fitted by a tri-exponential decay from three excited states ES_1_, ES_2_, ES_3_ populated sequentially, with a single ES spectrum^23,24^. Both a sequential model, in which each short-lived excited state relaxes into the next longer-living state, and a parallel model, in which the excited states decay independently, give an equally good fit to the data. It is therefore up to now unclear whether the different time constants and their different reactivity are representative of a relaxation process in the excited state (i.e., an evolution from a more- towards a less-productive minimum on the excited state surface), or whether they are due to heterogeneity or inhomogeneity, due to small structural differences between proteins^36,37^.

**Fig. 5:**
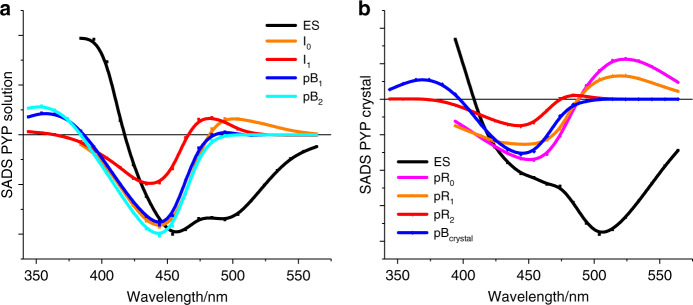
UV–Vis SADS of PYP. The SADS in solution (**a**) and crystalline (**b**) form have been resolved from fitting the target model shown in Fig. 4 to the experimental data, and display the bleached ground state, and stimulated emission, as negative features and induced absorption as positive features. The characteristic blue (≈370 nm) absorption of pCA in the pB states signals protonation of pCa. The region of the ground state bleach in the PYP_C_ dataset appears to be diminished with respect to that in PYP_S_; this is an effect of the high absorption of the crystals around 444 nm. Note that data from Yeremenko et al.^19^ has been added to the analysis to more reliably estimate the dynamics and SADS on the ~10–300 ms timescale and to span the full photocycle dynamics up to 1 s. Source data are provided as a Source Data file.

Each ES can decay into the ground-state intermediate (GSI), and via that return to the ground state, and into the isomerized pR_0_/I_0_ state(s). GSI is a short-lived component, which decays into the stable ground state on a picosecond time scale without giving rise to formation of (a) transient photocycle intermediate(s)^23,35^. The UV–Vis species associated difference spectra (SADS) that result from this analysis are shown in Fig. 5. The I_0_, I_1_, pR_0_, pR_1_, pR_2_ intermediates display red shifted product absorption, typical for the pCa *cis*-isomer, whereas the pB_1_, pB_2_, pB_crystal_ intermediates display blue-shifted product absorption, typical for the protonated pCa (*cis*-isomer).

**Fig. 6:**
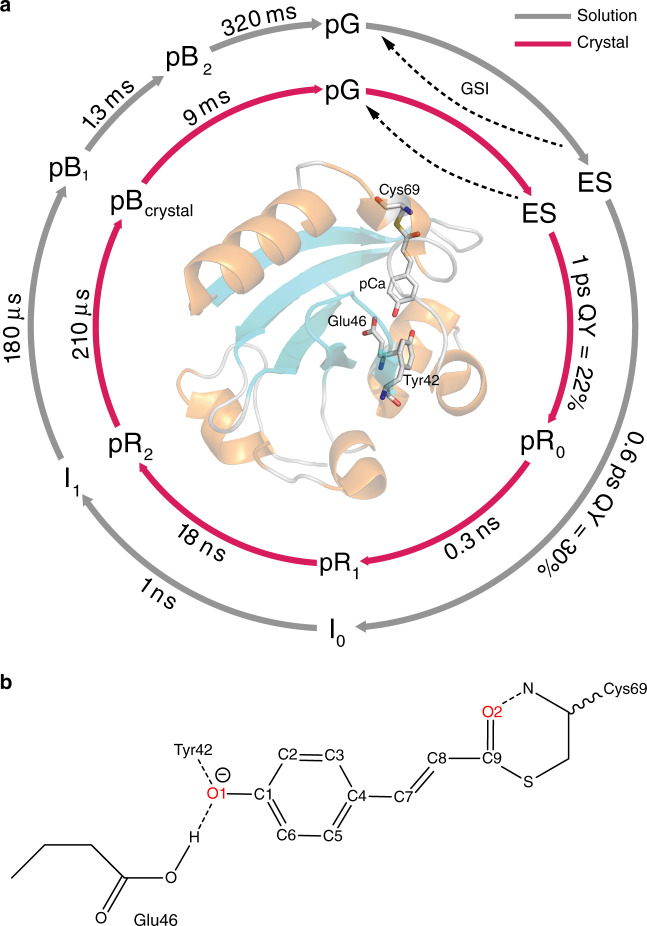
Photocycle scheme of PYP in solution and in crystalline form and pCa structure. **a** Simplified photocycle scheme for PYP_C_ (red arrows) and for PYP_S_ (gray arrows). The full kinetic schemes for PYP_S_ and PYP_C_ are shown in Fig. 4. Upon absorption of light by pG, the excited state is formed (ES), from which pCa trans-to-cis isomerization takes place in 0.6 ps in PYP_S_ and ~1 ps in PYP_C_, with quantum yields of 30 and 23% respectively. In PYP_C_, an additional intermediate is formed, pR_0_, followed by pR_1,2_ respectively, in comparison to the intermediates (I_0,1_) in PYP_S_. **b** pCa molecular structure in trans-configuration and interactions with neighboring sidechains. Hydrogen bonds are indicated by dashed lines.

In accordance with the observed differences in the time traces, fitting of the data against the target model yields different rates for PYP_S_ and PYP_C_, and an extra intermediate was required for PYP_C_. The initial decay of the ES in PYP_C_ is fastest, 0.35 ps, but results in only 10% pR_0_. A second decay phase in the ES leads to further pR_0_ formation in 2.6 ps, resulting in an overall quantum yield of 23% (Fig. 4). For clarity, we also present a simplified version of the photocycle in Fig. 6. There, we indicate the excited state process of PYP_C_ with a weighted average of 1 ps. In PYP_S_, 29% of I_0_ is formed in 0.6 ps and another 2% in 2 ps. We summarize this as, formation of the first *cis* isomer of pCa, with red-shifted product absorption, is slower in PYP_C_ than in PYP_S_ (1 vs 0.6 ps) and is formed with a lower quantum yield (0.23 vs 0.31). In the formation and dynamics of the initial red-shifted *cis*-isomer photoproducts in PYP_C_ an additional intermediate is present: pR_0_, pR_1_, pR_2_ vs I_0_ and I_1_ in PYP_S_. pR_0_ has a shorter lifetime than I_0_ (0.3 vs 1 ns) and pR_1_ decays into pR_2_ in 18 ns. The formation of pB-like states occurs on similar timescales in PYP_S_ and PYP_C_, but with a lower quantum yield, as only half of the pR_2_ states form pB_C_, resulting in a pB yield of 0.3 vs 0.11 in PYP_S_ and PYP_C_ respectively. Recovery of the ground state takes 9 ms in PYP_C_ and occurs with biphasic time constants of 1.3 and 320 ms in PYP_S_^19^.

The PYP_S_ photocycle kinetics are in agreement with earlier spectroscopic reports^19,23–25,27,28,35,38–45^, while the PYP_C_ results closely follow those observed in TRC by Schotte^2^, Jung^3^ and Ten Boer et al.^11^ (Note that these latter three studies mutually agree on the data, but differ in the bond-order of the double bond that can isomerize in the chromophore, due the use of DFT optimized structure vs non-DFT optimized structures^46^. In a recent ab initio computational study, the former structure was favored^47^). The agreement between the current data and those of TRC includes the dynamics on the 18 ns timescale associated with formation of pR_2_, 100% yield of pR_2_ from pR_0_^2^ and the 22% overall pR yield^11^, though the former reported the pR_2_ to pB transition to take place in 410 ms. The results for PYP_S_ and PYP_C_ clearly demonstrate an effect of the crystallization on the PYP photocycle, including on the earliest photocycle events.

### Assignment using site-specific isotope labeling in mid-IR

**Fig. 7:**
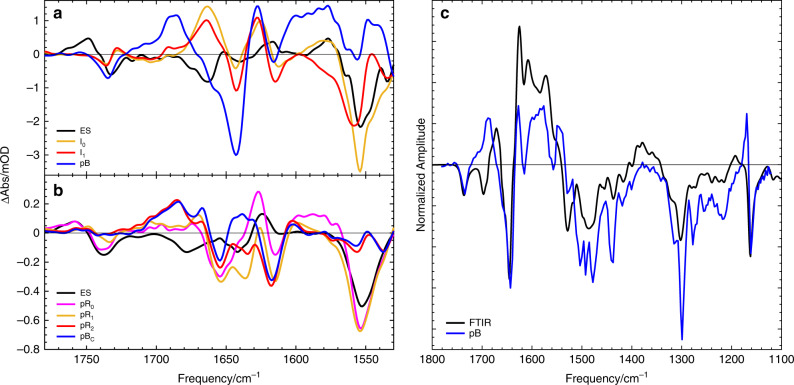
Species-associated-difference spectra (SADS) in the mid-IR spectral range. The SADS have been resolved from fitting the target model shown in Fig. 4 to the experimental data, and display the bleached ground state as negative feature and product state absorption as positive feature. **a** SADS of PYP_S_, **b** SADS of PYP_C_. **c** An accumulated PYP light-minus-dark difference FTIR spectrum and the SADS of the pB state of PYP in solution (also shown in **a**). Absorption changes in the 1740–1760 cm^−1^ region report on the hydrogen bond interaction between Glu46 and the phenol ring of pCa: higher frequency indicates weakened bond, lower frequency a stronger bond; Absorption changes in the 1690–1630 cm^−1^ region report on carbonyl stretches with a similar effect of a hydrogen bond on its frequency. The 1555 cm^−1^ band has been assigned to pCa C = C stretch and phenol ring modes^50,51,53^, see also Supplementary Table 1.

**Fig. 8:**
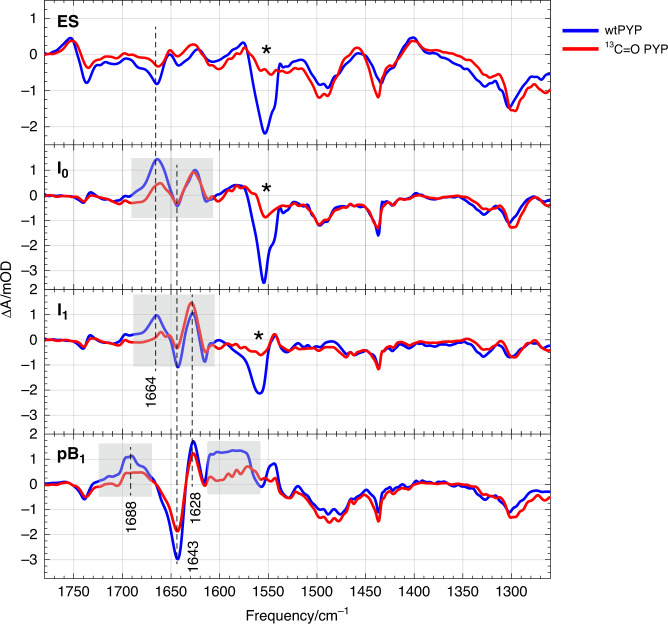
Mid-IR SADS of wild type and isotope-labeled PYP at position C9. WT-PYP (blue) and ^13^C = O - PYP (red) SADS of the ES, I_0_, I_1_, pB_1_ states are shown in the region between 1780–1250 cm^−1^. In the region 1540 to 1250 cm^−1^ the labeled PYP spectrum was scaled by a factor 0.5 for better comparison with the unlabeled spectra, whereas from 1780 to 1540 cm^−1^ no scaling was performed. The differences induced upon labeling of the carbonyl of pCa of PYP with ^13^C are highlighted with colored boxes in the graph and systematic losses around 1555 cm^−1^ bleach is shown by a star (*) symbol.

The SADS in the mid-IR spectral range of PYP_S_ and PYP_C_ (Fig. 7) allow for a more detailed structural analysis of the photocycle intermediates. Of particular interest is the timescale of the breaking of the hydrogen bond between pCa carbonyl and the Cys69 residue and its role in facilitating the isomerization process^2,3,11,35^. However, the frequency of pCa carbonyl in the I_0_ state has been a matter of contention^35,48^. To improve the mid-IR spectral assignment, we utilized a ^13^C isotopic label at position C9 of the pCa chromophore (^13^C = O pCa– PYP, see atom numbering in Fig. 6b). In doing so, the pCa carbonyl mode is expected to downshift, in addition to all modes involving C9, following earlier Raman analysis^49,50^. Unlabeled PYP and ^13^C = O – labeled PYP samples in solution were measured consecutively in a single experiment and repeated twice to ensure reproducibility. Both datasets were modeled according to the schemes described above, with all time constants fixed. In Fig. 8, we report the mid-IR SADS of both samples, where positive bands originate from product-state absorption due to the formation of the PYP photoproducts: ES, I_0_, I_1_, pB, and negative bands are due to the disappearance of ground state bands. The unlabeled SADS are consistent with those previously reported^35,38,39^. In Supplementary Table 1, we summarize the assignments of the spectral features observed in Figs. 7 and 8 that can be made based on vibrational spectroscopies and DFT calculations of the pCa chromophore, various isotope-labeled and point mutants of PYP previously reported^35,39,44,45,49–54^.

Comparison of the unlabeled and labeled ^13^C = O PYP_S_ spectra reveals a large isotope effect for the 1664/1643 cm^−1^ positive/negative bands appearing in the I_0_ and I_1_ SADS and at 1688/1643 cm^−1^ in pB_1_ (Fig. 8). This confirms that this feature is due to the pCa carbonyl, with the upshift reflecting breaking of the hydrogen bond between the pCa carbonyl and the protein upon isomerization. Note that part of the 1664/1643 cm^−1^ band shows no isotope effect and must therefore arise from Amide I C = O oscillators of the protein backbone. In addition, a large decrease in the amplitude of the 1554 cm^−1^ band is observed for the ES, I_0_ (1558 cm^−1^), I_1_ states. This is in agreement with the assignment to the C7 = C8 mode (Supplementary Table 1), and the observation of this band to be reduced in amplitude and downshifted by ~5–10 cm^−1^ in ^13^C = O—PYP ground-state Raman spectra^50,55^.

### Structural changes occurring during the photocycle of PYP_S_ and PYP_C_

The mid-IR SADS of PYP_C_ in Fig. 7 are qualitatively similar to those of PYP_S_, however, they exhibit several differences in quantitative details: in the pCa carbonyl region, a negative double band structure at 1655 and 1635 cm^−1^ in pR_1_ and pR_2_ is indicative of heterogeneity in the pCa–Cys69 hydrogen bond strength in the ground state. In the pR_0_ state, the 1660–1700 cm^−1^ region shows no positive band, suggesting that the hydrogen bond of the pCa carbonyl with Cys69 is intact in this intermediate. This is consistent with TRC structures reported for pR_0_ where pCa is highly contorted with its carbonyl oriented ≈90° out of plane with the phenolate, and the hydrogen bond intact^2,3^. In pR_1_, a minor positive band at 1670 cm^−1^ has appeared, signaling breaking of the hydrogen bond on the 0.3 ns timescale in at least a subset of the protein molecules (Figs. 2 and 7). The pR_2_ spectrum, formed in 18 ns, shows a band at 1688 cm^−1^, very similar to that of pB of PYP_S_. Note, however, that in PYP_S_ this band is formed only after 180 ms in pB (see corresponding time trace in Fig. 3). A shift of the free pCa carbonyl band from 1664 cm^−1^ in I_1_ to 1688 cm^−1^ in pB, assigned with the help of the isotopic labeling (Fig. 8), we suggest is due to the pCa carbonyl residing in a different environment, possibly more hydrophobic in pB compared to I_1_. Note that calculations on vibrational frequencies of PYP^49^ predict the pCa carbonyl to be at the relatively high frequency of 1688 cm^−1^ for the blue-shifted protonated product.

Changes in the region of the protonated carboxyl of Glu46 (1740–1760 cm^−1^) in PYP_C_ indicate that the hydrogen bond between the pCa phenol ring and Glu46 is relaxed throughout the photocycle of PYP_C_, in agreement with the reported lengthening from 2.50 to 2.94 Å in the TRC study of Pande et al^5^, but in contrast to the observed strengthening in the solution PYP_S_ I_0_ and I_1_ states^35,38^. The absorption changes in the Glu region disappear in pR_2_ and pB in PYP_C_, suggesting that the ground state configuration of Glu46 is recovered and that it remains protonated, even though the observed blue-shift of the chromophore electronic absorption in the PYP_C_ pB state signals chromophore protonation. This observation suggests that the proton donor to pCa in PYP_C_ is not Glu46. Indeed, Schotte et al.^2^ reported that in pB, Arg52 switches to an “open” conformation exposing the pCa phenolate and facilitating its protonation with a water molecule hydrogen-bonded to the phenolate and the protein backbone. Note that in the mid-IR study the timescale extended out to 0.3 ms; therefore, the protein structural changes associated with formation of the signaling state in PYP_S_ are not included. Hence, some further spectral differences associated with the signaling state can be observed when comparing the PYP_S_ pB_1_ spectrum with a difference spectrum collected in a steady-state FTIR spectrometer using light-on/light-off accumulation (Fig. 7c).

## Discussion

We conclude that our transient spectroscopic measurements on PYP_C_ are remarkably consistent with the photocycle events as observed in the TRC experiments of Schotte^2^, Jung^3^ and Ten Boer et al.^11^. Nevertheless, these dynamics differ from PYP dissolved in solution from the initial stages of the photocycle. The question arises of which transient structures the I_0_ and I_1_ intermediates in solution represent. Given the upshifted pCa C = O band in I_0_, indicating a broken hydrogen bond with Cys-69 following isomerization about the C7 = C8 double bond, it seems reasonable that this intermediate shares many structural properties with the pR_1_ intermediate observed in TRC and in the transient IR experiments. It should be noted, however that in pR_1_, the hydrogen bonds with Glu-46 are relaxed^2^ (Fig. 7b), while in I_0_ they are strengthened (Fig. 7a), which implies that the position of pCa relative to its binding pocket must be slightly different under the two conditions. Even so, I_0_ likely represents a fully isomerized, quasi-pR_1_ state formed immediately in 600 fs, while in the crystal the concurrent pR_0_ state represents an incomplete isomerization that remains stalled for 0.3 ns before isomerization is completed. We conclude that a pR_0_-like intermediate is never formed in solution. In the crystal, the pR_2_ intermediate evolves from pR_1_ through a *syn-anti* rotation of the C8-C9-S bonds to relieve steric strain of the latter pCa structure^2,3^. Such a process is likely to occur in the solution phase as well, so the I_1_ intermediate probably represents a quasi-pR_2_ structure. Such *syn-anti* rotation occurs significantly faster in solution (1 ns) than in the crystal (18 ns). Again, we note that the patterns of hydrogen-bond relaxation or strengthening with Glu-46 are reversed between crystal and solution for I_1_ and pR_2_ (Fig. 7a, b).

An overall picture emerges, where in solution, a rapid succession of isomerization and rotary motions finally result in a structurally relaxed, red-shifted pCa chromophore on a 1 ns timescale. In the crystal, similar but not identical motions take place that are significantly delayed with respect to solution and where pR_0_ represents a temporarily arrested isomerization. Considering the early onset of the diverging structural dynamics and their localized nature, the confining effects of crystal packing may be less likely the cause of our observations. In the Yeremenko study^19^ the effect of the presence of other solutes in the crystallization buffer, i.e., PEG 2000, which is used as a precipitant in growing crystals, was investigated. Increasing the PEG2000 concentration to the limit of solubility (200 g/L) was found to have no effect on PYP_S_ dynamics. A difference in pH (8 in solution versus 6 in the crystal) also cannot be the cause, as the PYP_S_ dynamics until 5 ns have been shown to be independent of pH in a range between 5 and 10^31^, which we confirmed here by recording the dynamics at both pH 6 and 8 (see Supplementary Fig. 1). The decreased level of protein hydration in the crystalline phase with respect to that in solution may be the more likely cause for the observed differences between crystal and solution. Overall lower hydration in the crystalline state may lead to a lower water concentration throughout the protein scaffold including the pCa binding pocket and altered electrostatic interactions. The influence of dehydration on the later part of the photocycle of PYP has been studied in films with low hydration levels^56^ and cell-mimetic environments^57^. Dehydration was found to alter both the mechanism and the kinetics of the later parts of the photocycle significantly^56,57^. On earlier timescales, the isomerization quantum yield and rate have been found to be sensitive to the arrangement of water molecules inducing altered hydrogen bond interactions in the PYP active site^28^. However, the molecular basis of the different ultrafast parts of the photocycle of PYP_C_ and PYP_S_, whether this is dehydration, altered viscosity or confinement by the crystal lattice remains to be decided and it may well be a mixture of these factors.

Another effect often considered when comparing spectroscopic and TRC results, is the excitation density, which is typically high in TRC experiments, to achieve a high population of the photocycle intermediates. A recent report focused on the comparison of 0.25–10 ps dynamics of bacteriorhodopsin in membranes and crystalline form, investigating the effect of the high power excitation regimes employed in TRC experiments on the dynamics^14^. Here, we used a low excitation density for both cases, to avoid multi-photon processes. Remarkably, the spectroscopic results on crystalline PYP could be well linked to those of the various TRC experiments, suggesting that in PYP high(er) excitation densities overall lead to a very similar photocycle. Overall, these previous accounts and ours above strongly suggest that transient crystallographic and spectroscopic techniques are highly complementary and most effective when applied in a symbiotic fashion in the context of resolving of protein dynamics with varying sample treatment, in order to relate to the dynamics in-vivo.

## Methods

### Protein expression and purification

wtPYP and ^13^C = O – labeled PYP were produced by reconstituting apo-PYP with the 1,1′-carbonyldiimidazole derivative of *p*-coumaric acid chromophore^58,59^ For experiments in aqueous solution the reconstituted holoproteins were purified by using an Äkta FPLC system (GE Healthcare) in two subsequent steps: Ni-affinity chromatography and anion exchange chromatography, respectively.

Reconstituted PYP in cell free extract^53^ was loaded on two Ni-affinity columns (HisTrap FF, GE Healthcare), washed with 10 column volumes of buffer A (20 mM Na_2_HPO_4_, 20 mM imidazole, 150 mM NaCl pH =7.5), and eluted with a gradient with buffer B (20 mM Na_2_HPO_4_, 500 mM imidazole, 150 mM NaCl, pH = 7.5) from 0 to 100% in 10 column volumes. The fractions containing PYP were then dialyzed overnight against 20 mM Tris pH = 8.0 and further purified with anion exchange chromatography (HiTrapQ HP columns) with a gradient of buffer A (20 mM Tris, pH = 8.0) and buffer B (20 mM Tris plus 1 M NaCl, pH = 8.0), after filtering of the protein solution (0.2 µm filter). After washing 10 column volumes with buffer A, a gradient of 0 to 12.5% B in 10 column volumes and subsequently a gradient of 12.5 to 20% B in 3 column volumes was used. After that the column was regenerated with 100% B.

To crystallize PYP an extended purification protocol was used: The purified protein was first dialysed to 20 mM Tris buffer pH = 8 and concentrated to >20 mg/ml with a 5.000 MW spin concentrator. The N-terminal poly-histidine tag of the protein was then removed via overnight cleavage with Enterokinase (1.000:1 (w/w); Sigma-Aldrich) at 37 °C. Non-digested protein was removed via Ni-affinity chromatography (see above) and the holo-PYP collected from the flow-through fractions of the latter step was subjected to anion exchange chromatography (using a HiTrapQ HP column) with a gradient (see above) of buffers A (20 mM Tris pH = 8.5) and B (20 mM Tris, 1 M NaCl, pH = 8.5), again after filtering of the protein (see above). The fractions from this column with the highest PYP content were then checked for the dynamics of photocycle ground-state recovery at 464 nm, and for purity on SDS_PAGE, filtered, pooled, and concentrated >25 mg/ml with a 5000 MW spin concentrator.

The final purification step to produce PYP suitable for crystallization experiments was a gel filtration step with a Superdex 75 column in 100 mM MES, pH = 6.5 with ascending flow of 0.5 ml/min and the protein in the fractions with the highest purity was concentrated to ~25 mg/ml in 100 mM MES, pH = 6.5 with a 5000 MW spin concentrator.

Accordingly, the purified PYP protein was used for spectroscopy in aqueous solution without prior removal of the genetically introduced N-terminal poly-histidine tag, in 20 mM Tris buffer, pH = 8. For ^13^C = O – labeled PYP, the purification of ^13^C- over ^12^C-labeled protein was checked with mass spectroscopy and found to be 100%. Both unlabeled and labeled samples were pipetted in between two CaF_2_ windows (25 mm diameter, 2 mm thickness) separated by a 10 µm Teflon spacer, resulting in samples with OD_446_ = 0.8 /10 μm for both the visible and mid-IR experiments. All experiments were performed at room temperature. See Supplementary Fig. 3 for the steady-state UV-vis absorption spectrum.

### PYP crystallization protocol

Crystalline PYP in space group P6_5_ was prepared using the vapor diffusion method drop method according to published protocol^19^. In short, 4 µl of concentrated PYP solution (27.5 mg/ml) was combined with equal volume mother liquor (100 mM MES buffer pH 6.5, 40% PEG 2000) on a circular microscope coverslip. This suspension was carefully fixed over a compartment of a 24 well plate containing 1 ml mother liquor and sealed with vacuum grease. The crystallization proceeded for 6 days at 21 °C and crystals were then harvested using a micropipette. The resulting product was washed with fresh mother liquor and stored at 4 °C. For transient absorption measurements, crystals were loaded between two CaF_2_ windows without a spacer and crushed for optimal transparency, similar to the procedure in ref. ^60^. A minimal amount of scatter was observed cf, Fig. 1. The OD_446_ was ~0.8 but varied widely within the sample. A D_2_O mother liquor was used for the mid-IR experiment to reduce background buffer absorption.

### Transient electronic absorption spectroscopy

Dual regeneratively-amplified laser systems (Legend and Libra, Coherent, Santa Clara, CA) seeded by a common Ti:Sapphire oscillator generated <50 fs pulses at 800 nm with 1 kHz repetition rate^61–63^. The Legend was used as a pump source for an optical parametric amplifier (OPerA Solo, Coherent) to produce 475 nm light with 15 nm bandwidth FWHM. A broadband supercontinuum probe beam was generated by focusing ≈5% of the Libra output onto a translating CaF_2_ plate. The pump (140 nJ) and probe beams were temporally and spatially overlapped on the sample. The pump beam was focused to a spot with a FWHM of 250 µm, and the probe beam to 125 µm. The probe was spectrally dispersed and detected with a multichannel detection system (Entwicklungsburo Stresing) comprised of a 1024 pixel back-thinned FFT-CCD detector (S7030-1006, Hamamatsu). The transient absorption signal was measured in situ by modulation of the pump beam with an optical chopper at one-half the laser repetition rate. An excitation intensity of 400 nJ per pulse was used for both samples. In combination with the off-center excitation wavelength of 475 nm this resulted in an excitation density of ~5% excited proteins. This value was carefully chosen to avoid multi-photon processes and/or photoionization^20,60^. The polarization of pump and probe beams was under the magic angle. In combination with the crushing procedure of the crystals, this minimizes possible photo-selection effects in the crystals.

### Transient mid-IR absorption spectroscopy

Transient absorption data in the mid-IR spectral region were recorded with a similar setup utilizing dual femtosecond Ti:Sapphire amplifier systems seeded by a common oscillator (MaiTai, Spectra Physics). The first (1 kHz, Hurricane, Spectra Physics) produced ≈85 fs pulses at 800 nm with 0.6 mJ pulse energy which was used to pump an OPA (TOPAS, Light Conversion) tuned to 475 nm for electronic excitation. The output of the second amplifier (1 kHz, Spitfire Ace, Spectra Physics) pumped a separate OPA (TOPAS-C, Light Conversion) to produce near-IR signal and idler beams which then underwent subsequent difference frequency mixing in AgGaS_2_ to yield ≈100 fs mid-IR pulses from 1–10 µm with ≈200 cm^−1^ bandwidth FWHM. The IR probe beam was focused on the sample with a 10 cm CaF_2_ lens (125 µm spot diameter) and spatially and temporally overlapped with the excitation beam (250 µm spot diameter) in the sample plane. The emerging probe beam was dispersed in a grating spectrograph and detected with a liquid nitrogen-cooled 64 element Mercury Cadmium Telluride photodiode array (Infrared Associates). The setup was contained in a box continuously purged with dry air to minimize ambient water vapor absorption. An excitation intensity of 1 µJ per pulse was used for both samples. In combination with the off-center excitation wavelength of 475 nm this resulted in an excitation density of ~10% excited proteins. This value was sufficiently low to avoid multi-photon processes and/or photoionization^20^. The polarization of pump and probe beams was under the magic angle. In combination with the crushing procedure of the crystals, this minimizes possible photo-selection effects in the crystals.

### Data collection and analysis

The transient absorption data was collected as previously described^61,64^. In short, probe delays up to 6 ns were achieved with an optical delay line and long pump-probe delays (from 12 ns to 500 ms) by systematic adjustment of the pump laser Pockels cell timing. The solution sample was refreshed using a home-built Lissajous scanner, whereas the crystalline sample was continuously scanned in a linear pattern to prevent photodamage and allow for adequate dark state recovery during data collection. The integrity of the sample was monitored by measuring its linear absorption spectrum before and after data collection. For the mid-IR experiments, data was collected in the range of 1540–1780 cm^−1^. Labeled and unlabeled samples were measured consecutively and repeated to ensure reproducibility. Calibration of the spectrometer was done using polynomial fitting of vibrational bands of known reference samples. This procedure had an uncertainty of 4 cm^−1^, therefore we used the results from the FTIR measurements to correct the time-resolved IR spectra by applying up to 4 cm^−1^ shifts. The transient absorption data were analyzed by global analysis using the Glotaran software package^65^. Details of this method and analysis of the data are described in the section below.

### Target analysis

To extract the pure species-associated spectra, we applied a target model providing a physical description of the photocycle of PYP. Pump-dump-probe studies, previous transient absorption studies^23,24,26–28^ were used to confine our target model. With this method, we can extract the characteristic spectra of all photocycle states, namely, contributions from the excited state (ES), primary photoproduct (I_0_), and subsequent intermediates. The photocycle scheme and the corresponding parameters for the kinetic model are shown in Fig. 4.

### Reporting summary

Further information on experimental design is available in the Nature Research Reporting Summary linked to this paper.

## Supplementary information

Supplementary Information

Peer Review File

Reporting Summary

## Data Availability

Source data are provided as a Source Data file. Other data are available from the corresponding author upon reasonable request. Source data are provided with this paper.

## Source data

Source Data
