# Supplementary material for: Confinement in crystal lattice alters entire photocycle pathway of the Photoactive Yellow Protein
Source: Nat Commun. 2020 Aug 25;11:4248. doi: 10.1038/s41467-020-18065-9 (PMC7447820; doi:10.1038/s41467-020-18065-9)
Supplement: Supplementary file 1 — Supplementary Information [file 41467_2020_18065_MOESM1_ESM.pdf]

Supplementary Information

Confinement in crystal lattice alters entire photocycle pathway of the Photoactive Yellow Protein

Patrick E. Konold, Enis Arik et al.

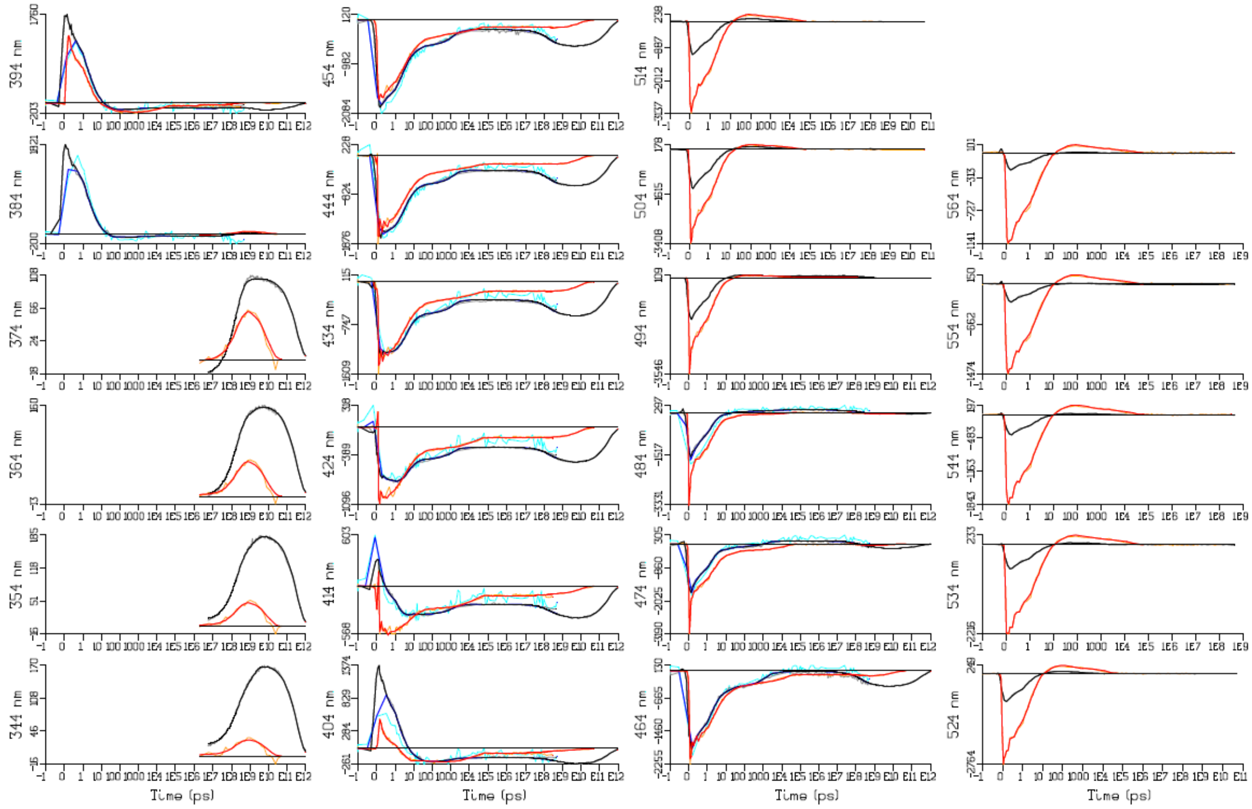

Supplementary Figure 1. Selected time traces of the UV-Vis difference absorption of Photoactive Yellow Protein in solution at pH 8 (black), solution at pH 6 (cyan) and in the crystal (red). Light colors indicate the data, whereas the dark colors indicate the fit. In the study with sub-picosecond time resolution the latest time point was 0.31 milliseconds. The data taken from Yeremenko et al.[1] (with microsecond time resolution) have the latest time point at 48 milliseconds (crystal) or 1 second (solution). Wavelength is indicated in the ordinate label. Note that the time axis is linear until 1 picosecond (after the maximum of the IRF), and logarithmic thereafter. A single scaling parameter has been used to connect the data from the experiments with sub-picosecond and microsecond time resolution.

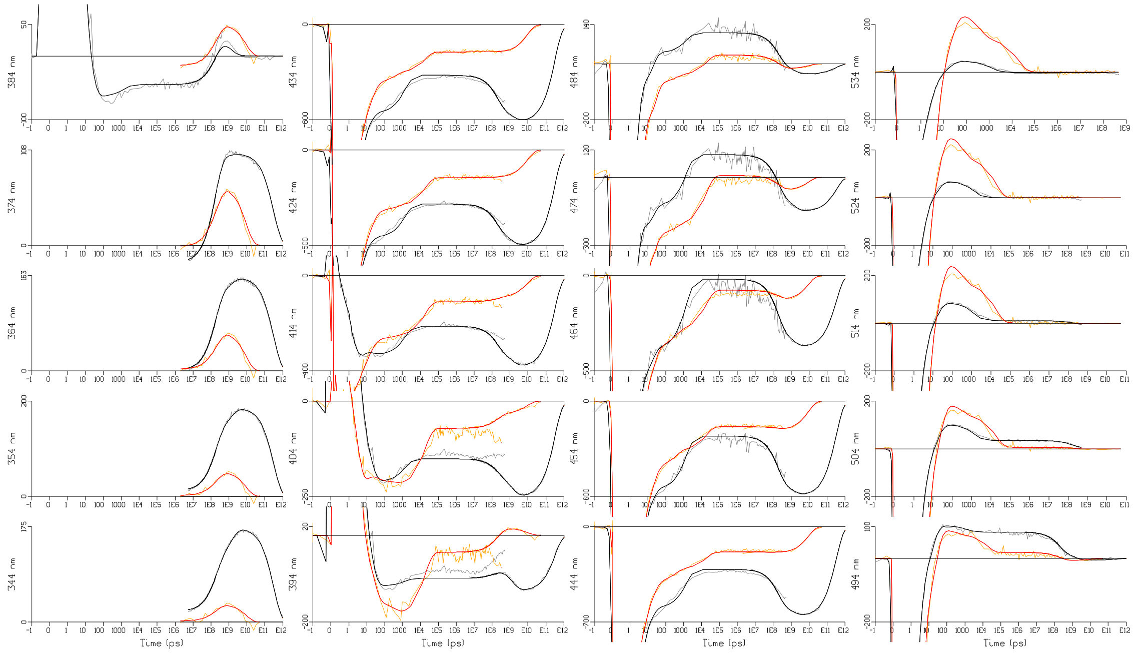

Supplementary Figure 2. Zoom-in of selected time traces of the UV-Vis difference absorption of Photoactive Yellow Protein in solution (black) or in the crystal (red) emphasizing the signals after the decay of the excited state. Light colors indicate the data, whereas the dark colors indicate the fit. In the study with sub-picosecond time-resolution study the latest time point was 0.31 milliseconds. The data taken from Yeremenko et al.[1] have the latest time point at 48 milliseconds (crystal) or 1 second (solution). Wavelength is indicated in the ordinate label. Note that the time axis is linear until 1 picosecond (after the maximum of the IRF), and logarithmic thereafter.

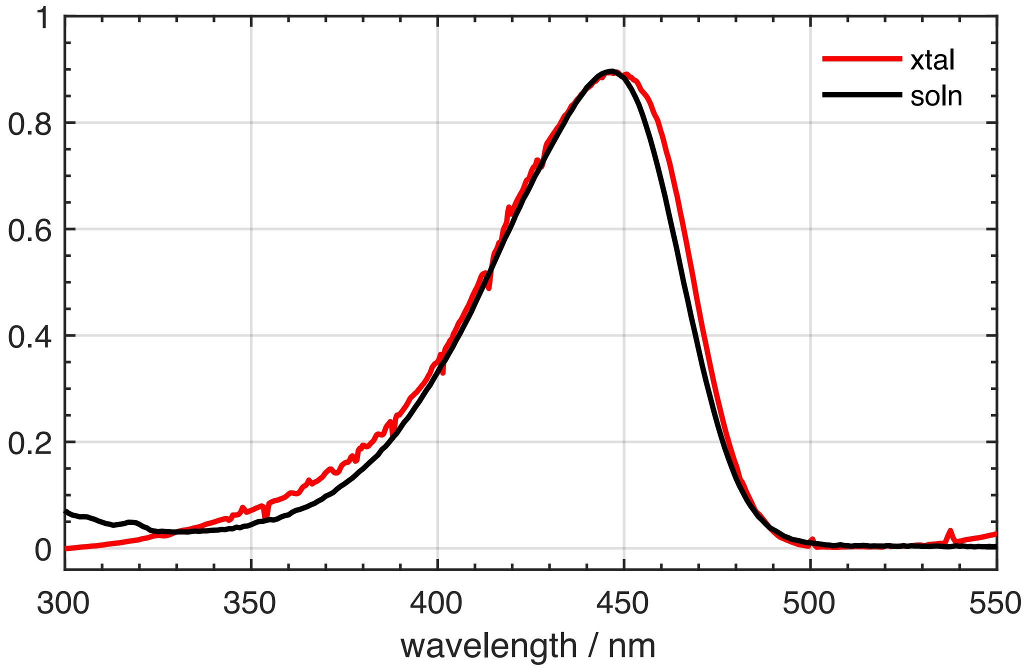

Supplementary Figure 3. Steady state uv-vis absorption spectrum of PYP in solution and in crystalline form.

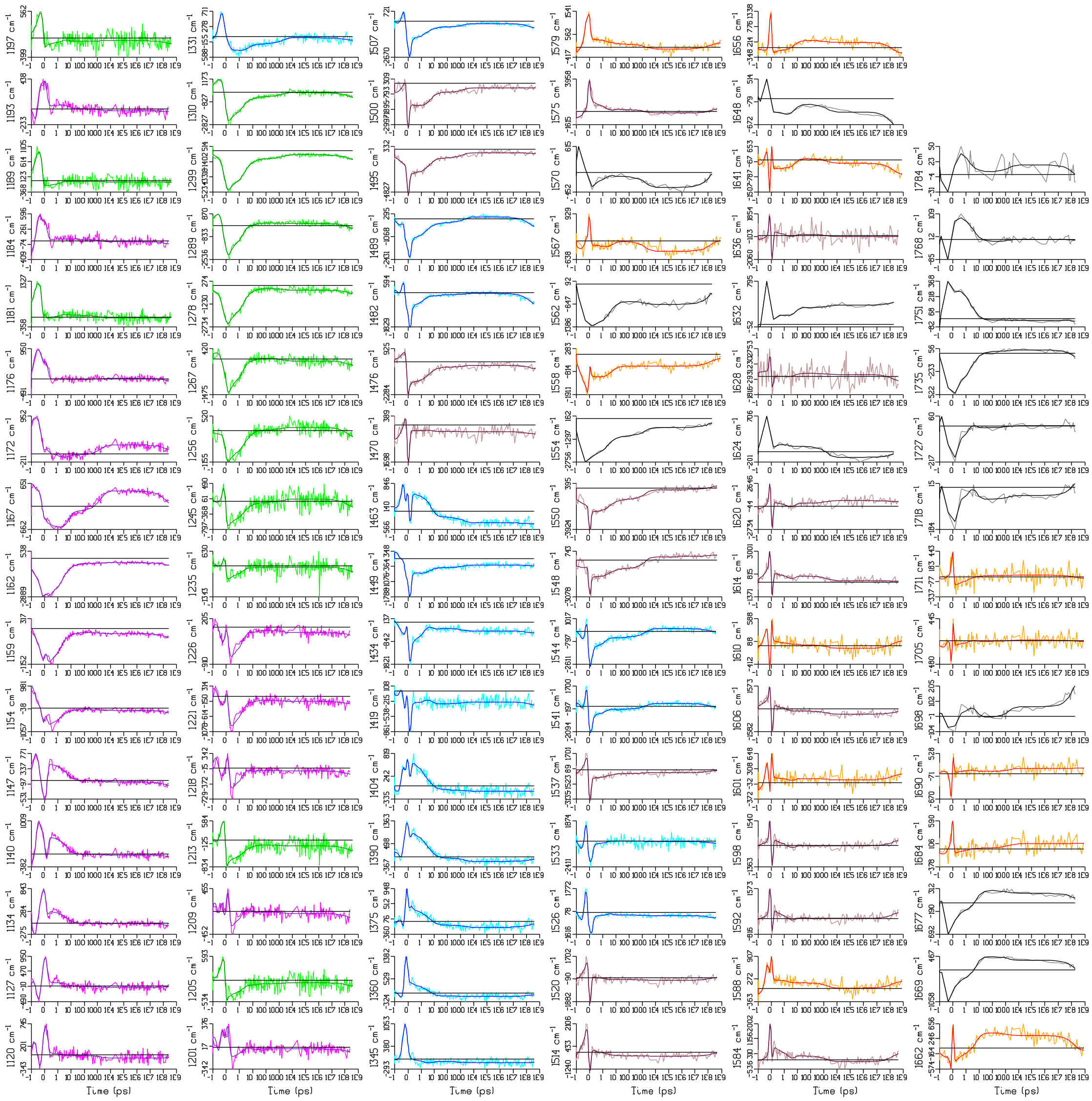

Supplementary Figure 4. Selected time traces of the difference absorption of PYP in solution in the mid-IR. Light colors indicate the data (with different colors indicating experiments in different wavenumber ranges), whereas the dark colors indicate the fit. Wavenumber is indicated in the ordinate label. Note that the time axis is linear until 1 picosecond (after the maximum of the IRF), and logarithmic thereafter.

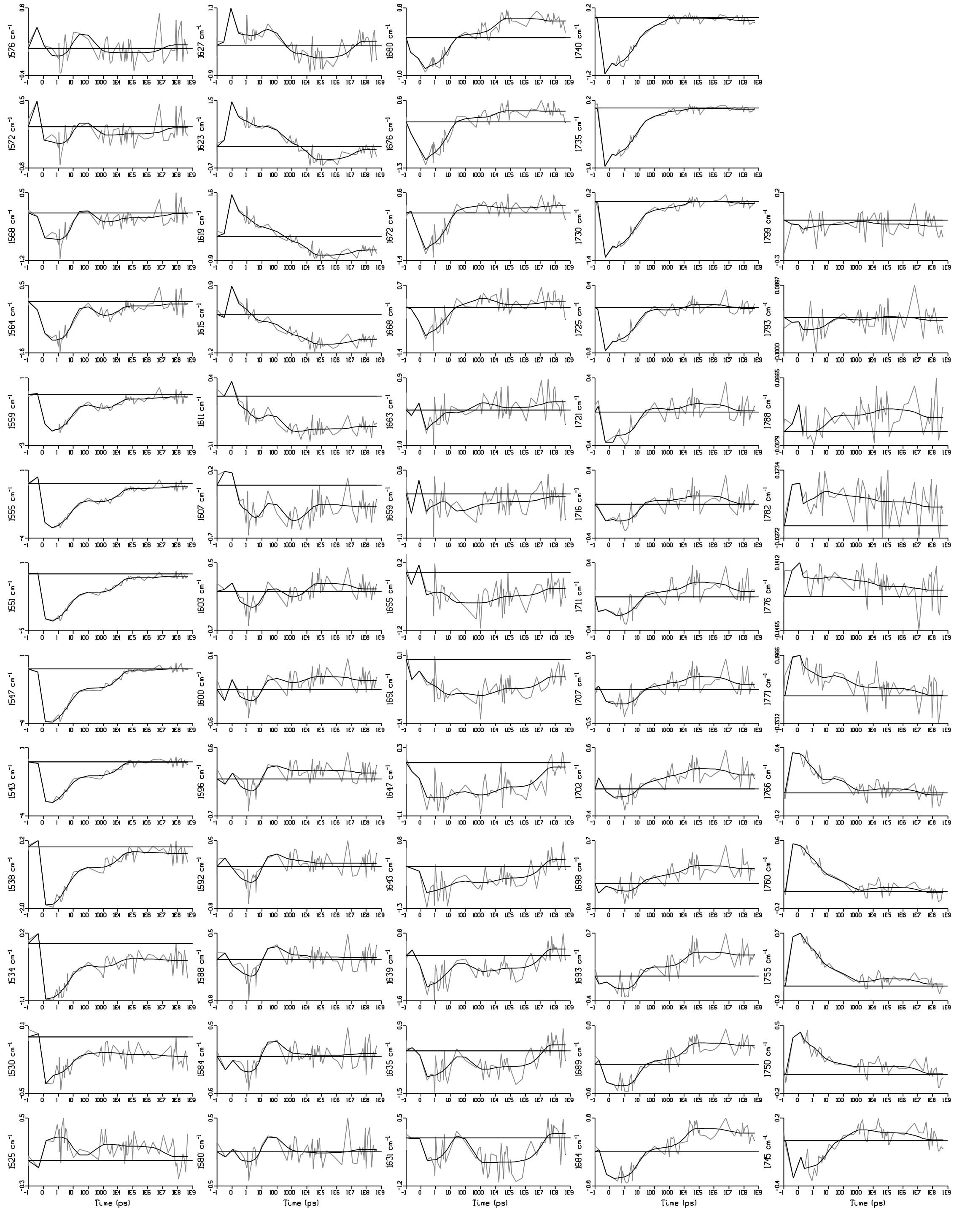

Supplementary Figure 5. Time traces in the midinfrared spectral range of the difference absorption of Photoactive Yellow Protein in the crystal. Grey indicates the data, whereas black indicates the fit. Wavenumber is indicated in the ordinate label. Note that the time axis is linear until 1 picosecond (after the maximum of the IRF), and logarithmic thereafter.

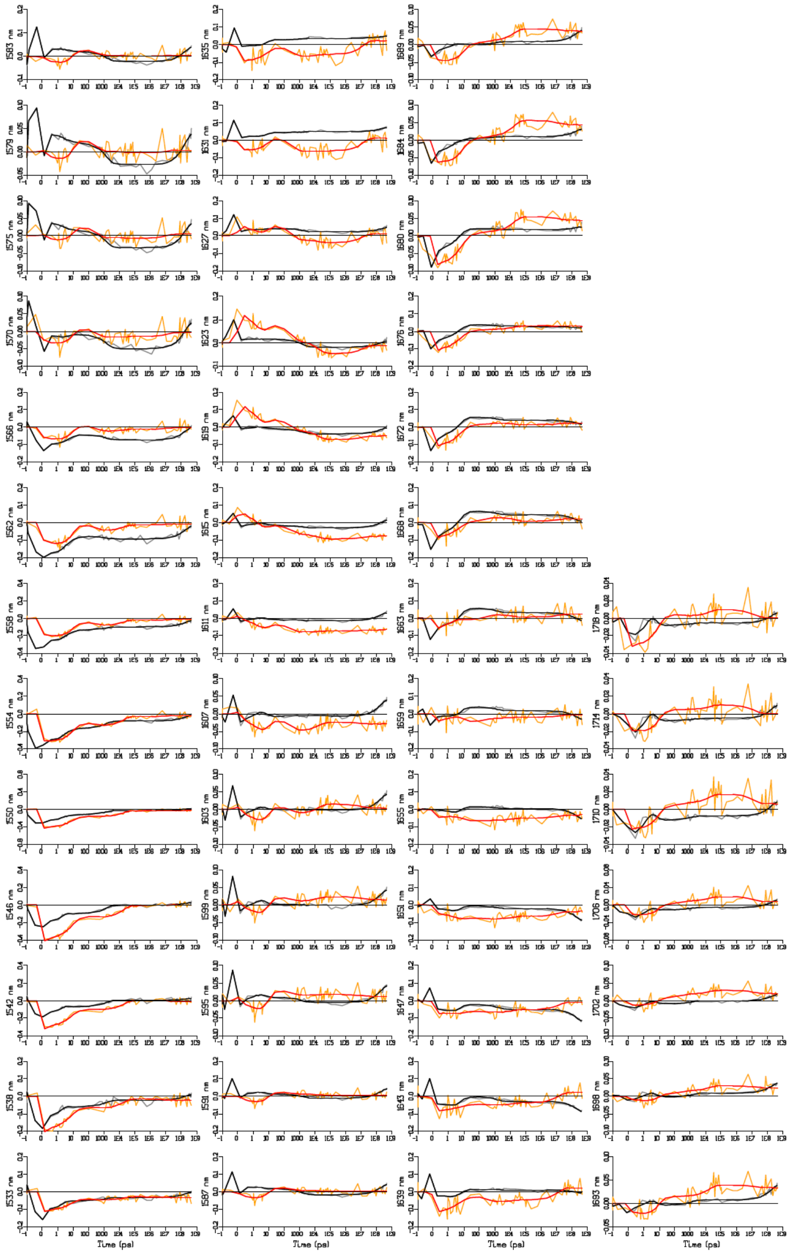

Supplementary Figure 6. Time traces in the midinfrared spectral range of the difference absorption of Photoactive Yellow Protein in the crystal (orange) and in solution (grey) overlaid. Wavenumber is indicated in the ordinate label. Note that the time axis is linear until 1 picosecond (after the maximum of the IRF), and logarithmic thereafter.

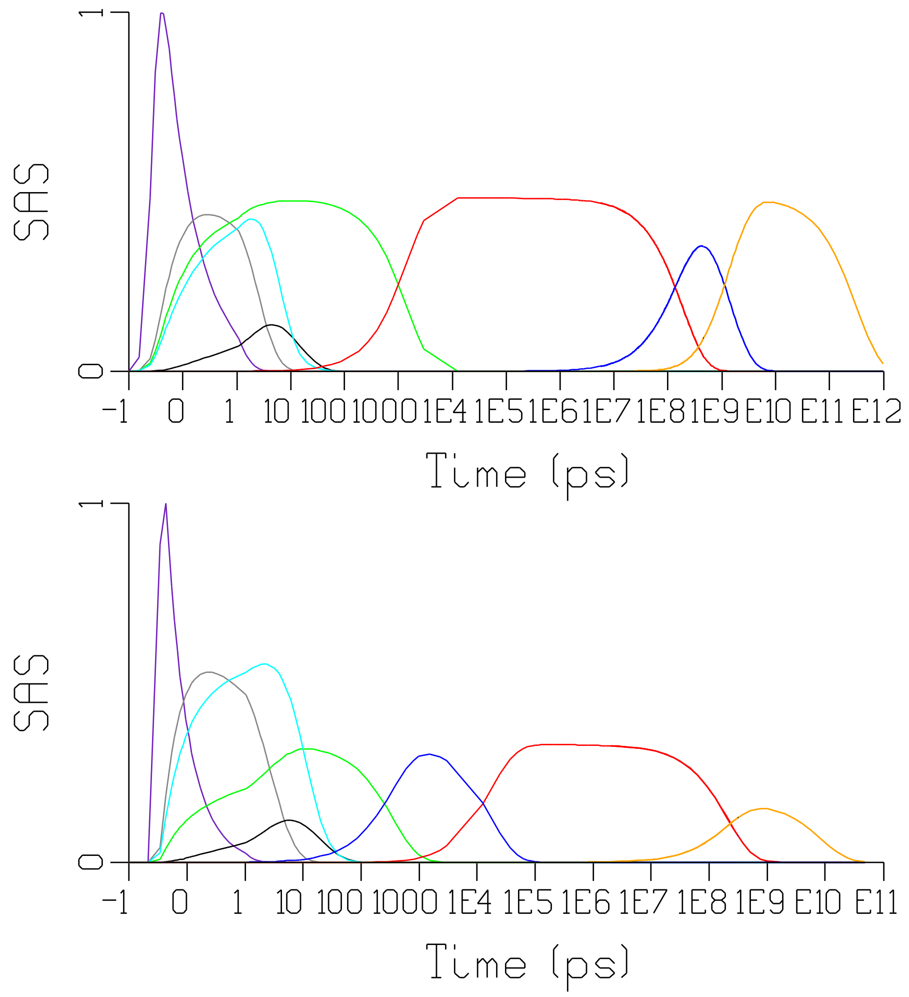

Supplementary Figure 7. Concentrations computed with the kinetic scheme of the photocycle of PYP in solution (top) and in crystalline form (bottom, from VIS data). Key: ES_{1-3}: purple, grey, black; GSI: cyan; I_0: green, I_1:red, I_2:blue, pR_0: green, pR_1:blue, pR_2:red, ;pB, $pB_{crystal}$: orange.

Supplementary Table 1. Band assignment to normal modes. Indicated parenthetically is the magnitude of shift or amplitude change observed upon $^{13}C9$ labeling.

ω_{obs} (cm^{-1})	Assignment	Intermediates	Reference
1738, 1748, 1729	C=O Glu46<br>Reflects weakening followed by strengthening of H-bond	GS, ES, I_0/I_1	[2-4]
1688	Arg52 in more hydrophobic location	I_2	[5,6]
1688 (loss)	pCa free C=O	I_2	This study
1664 (-33)	pCa free C=O	I_0, I_1	This study, [7]
1659	Protein backbone amide I	I_0, I_1	[8]
1643(loss)	pCa C=O + protein backbone amide I	GS,ES, I_0, I_1, I_2	This study, [9]
1558 (loss)		I_1	
1554 (loss)	pCa C=C + phenol ring (Y19a of tyrosine).	ES, I_0	
1545 (loss)		I_2	[9-11]
1500	Phenol ring (Y19a of tyrosine) + vinyl C7=C8 stretching	ES, I_0, I_1, I_2	
1440	Y19b of tyrosine		
1316 (-4), 1302 (-4)	aromatic ring (Tyr14) and $HC_7{=}C_8H$ rocking motions.	ES, I_0, I_1, I_2	

Supplementary References

1 Yeremenko, S., van Stokkum, I. H. M., Moffat, K. & Hellingwerf, K. J. Influence of the crystalline state on photoinduced dynamics of photoactive yellow protein studied by ultraviolet-visible transient absorption spectroscopy. *Biophysical journal* **90**, 4224-4235, doi:10.1529/biophysj.105.074765 (2006).

2 Brudler, R., Rammelsberg, R., Woo, T. T., Getzoff, E. D. & Gerwert, K. Structure of the I1 early intermediate of photoactive yellow protein by FTIR spectroscopy. *Nat Struct Mol Biol* **8**, 265-270, doi:10.1038/85021 (2001).

3 Xie, A., Hoff, W. D., Kroon, A. R. & Hellingwerf, K. J. Glu46 Donates a Proton to the 4-Hydroxycinnamate Anion Chromophore During the Photocycle of Photoactive Yellow Protein. *Biochemistry* **35**, 14671-14678, doi:10.1021/bi9623035 (1996).
4 Imamoto, Y. *et al.* Low-Temperature Fourier Transform Infrared Spectroscopy of Photoactive Yellow Protein†. *Biochemistry* **40**, 8997-9004, doi:10.1021/bi010021l (2001).
5 Tamm, L. K. & Tatulian, S. A. Infrared spectroscopy of proteins and peptides in lipid bilayers. *Quarterly reviews of biophysics* **30**, 365-429 (1997).
6 Xie, A. *et al.* Formation of a New Buried Charge Drives a Large-Amplitude Protein Quake in Photoreceptor Activation†. *Biochemistry* **40**, 1510-1517, doi:10.1021/bi002449a (2001).
7 Unno, M., Kumauchi, M., Sasaki, J., Tokunaga, F. & Yamauchi, S. Resonance Raman spectroscopy and quantum chemical calculations reveal structural changes in the active site of photoactive yellow protein. *Biochemistry* **41**, 5668-5674, doi:10.1021/bi025508o (2002).
8 Shingae, T., Kubota, K., Kumauchi, M., Tokunaga, F. & Unno, M. Raman Optical Activity Probing Structural Deformations of the 4-Hydroxycinnamyl Chromophore in Photoactive Yellow Protein. *J Phys Chem Lett* **4**, 1322-1327, doi:10.1021/jz400454j (2013).
9 Unno, M., Kumauchi, M., Tokunaga, F. & Yamauchi, S. Vibrational Assignment of the 4-Hydroxycinnamyl Chromophore in Photoactive Yellow Protein. *J. Phys. Chem. B* **111**, 2719-2726, doi:10.1021/jp066434j (2007).
10 Zhou, Y., Ujj, L., Meyer, T. E., Cusanovich, M. A. & Atkinson, G. H. Photocycle Dynamics and Vibrational Spectroscopy of the E46Q Mutant of Photoactive Yellow Protein. *The Journal of Physical Chemistry A* **105**, 5719-5726, doi:10.1021/jp004575u (2001).
11 Kim, M., Mathies, R. A., Hoff, W. D. & Hellingwerf, K. J. RESONANCE RAMAN EVIDENCE THAT THE THIOESTER-LINKED 4-HYDROXYCINNAMYL CHROMOPHORE OF PHOTOACTIVE YELLOW PROTEIN IS DEPROTONATED. *Biochemistry* **34**, 12669-12672, doi:10.1021/bi00039a024 (1995).
